# The Morbidity of Oral Mucosal Lesions in an Adult Swedish Population

**DOI:** 10.4317/medoral.19286

**Published:** 2013-06-22

**Authors:** Jairo Robledo-Sierra, Ulf Mattsson, Tage Svedensten, Mats Jontell

**Affiliations:** 1 PhD Student, Department of Oral Medicine and Pathology, Institute of Odontology, Sahlgrenska Academy, University of Gothenburg, Gothenburg, Sweden; 2 Associate Professor, Department of Oral Medicine and Pathology, Institute of Odontology, Sahlgrenska Academy, University of Gothenburg, Gothenburg, Sweden; 3 Private Dentist, Praktikertjänst Dental Care, Borås, Sweden; 4 Professor, Department of Oral Medicine and Pathology, Institute of Odontology, Sahlgrenska Academy, University of Gothenburg, Gothenburg, Sweden

## Abstract

Objectives: To study the severity of symptoms and estimate the prevalence of oral mucosal lesions in a non-referral adult Swedish population, as registered by general dental practitioners. This study also aims to evaluate the possibi-lity of dental practitioners collecting large quantities of reliable and accurate clinical data on oral mucosal lesions. Study Design: Data from 6,448 adult Swedish patients were collected by general dental practitioners using a standardized registration method. A correlation analysis between a group with oral mucosal lesions and a control group, with no oral mucosal lesions, was performed for various parameters such as symptoms from the oral mucosa, systemic diseases, medication, allergy history, tobacco habits and the patient’s own assessment of their general health. In addition, clinical photos were taken of all oral mucosal lesions in order to determine the degree of agreement between the diagnoses made by general dental practitioners and those made by oral medicine specialists. Results: A total of 950 patients (14.7%) presented with some type of oral mucosal lesion and of these, 141 patients (14.8%) reported subjective symptoms. On a visual analogue scale, 43 patients (4.5%) scored their symptoms <30, 65 patients (6.8%) scored their symptoms ?30, and 28 patients (2.6%) scored their symptoms ?60. The most debilitating condition was aphthous stomatitis and the most common oral mucosal lesion was snuff dipper’s lesion (4.8%), followed by lichenoid lesions (2.4%) and geographic tongue (2.2%). There was agreement between the oral medicine specialists and the general practitioners over the diagnosis of oral mucosal lesions on the basis of a clinical photograph in 85% of the cases (n=803). Conclusions: Nearly 15% of the patients with oral mucosal lesions reported symptoms. General practitioners could contribute significantly to the collection of large quantities of reliable and accurate clinical data, although there is a risk that the prevalence of oral mucosal lesions may be underestimated.

** Key words:**Epidemiology, oral mucosal lesions, oral medicine, examiner reliability.

## Introduction

The morbidity rate for oral mucosal lesions (OML) in the general population has not been thoroughly studied. Several OML are debilitating for patients and may also predispose to the development of life-threatening disorders. Although the morbidity rate for potentially malignant oral diseases has previously received some attention, little is known about the extent to which other lesions may represent a burden to society.

Patients’ own perceptions of their oral conditions are rarely assessed in epidemiological studies of OML. Studies of the impact of OML upon the Quality of Life (QoL) (1-3) have shown that patients are physically, socially and psychologically affected, and these effects tend to be underestimated by healthcare providers. It is therefore important to report the extent to which people suffer from symptoms and discomfort in the oral cavity.

Epidemiological data of OML derives mainly from lesion-specific studies that have been conducted on targeted or high-risk populations (4-11). A few studies have used random sampling methods in various geographical areas to identify the prevalence of OML in the general population (12-17). In these studies healthcare providers with a variety of professional backgrounds have performed the examinations of the oral mucosa, but the accuracy of such examinations has not been thoroughly assessed. Only a few reports have described in detail how the training of the practitioner was performed and evaluated.

It is therefore of interest to evaluate the accuracy of the registration by general dental practitioners (GP) of clinical information for large populations. New information technology makes it easier to involve GP in academic work of this kind and if their contributions are reliable, this could enable the expedient collection of extensive quantities of epidemiological data.

The aims were 1) to study the severity of subjective symptoms caused by OML and to assess the extent to which OML are a burden to society, 2) to estimate the prevalence of OML in a non-referral adult Swedish population, as registered by GP, and 3) to identify possible correlations between OML and general health.

## Material and Methods

-Patients

A total of 6,448 subjects, who visited their dentist between 2004 and 2006 for their annual examination, were invited to participate in the study. Of these, only one refused to participate because of dental fear. The patients were examined by one of six GP in six private dental clinics in Borås, a medium-sized town of 66,000 inhabitants in the Southwest of Sweden.

-Data collection

Prior to the study, all the participating dentists undertook training by an oral medicine specialist (MJ) in the diagnosis of OML, data collection and intraoral photographic techniques. The same electronic form, which was developed in MedView, was used to collect the clinical data in all cases. MedView is a computer system for the formalized registration and subsequent analysis of clinical and image-based information (18). It operates with an input application that is focused on the collection and computerised storage of clinical data. At the first examination, the medical history of each patient was recorded in MedView. The GP were asked to take clinical images of all mucosal lesions and all were equipped with the same type of camera (Sony Handycam 3 CCD). The resulting information was gathered in a single database and exported to MedVisualizer. This application is used for visualization and scanning of the information that is obtained from the database (18). Finally, the data selected for evaluation were transferred to Microsoft Excel for Mac 2011 for subsequent statistical analysis.

-Clinical information

The diagnostic labels and criteria for OML were in accordance with WHO, ICD-DA and with the modifications and complementary additions suggested by Axéll (12,19) and Axéll *et al.* (20). Systemic diseases were registered according to the International Classification for Diseases and Health Related Problems, 10th revision (ICD-10). Allergies reported by the patients were grouped into five different categories: “allergic to food substances”, “allergic to pollen/grass”, “allergic to animals”, “allergic to metals/chemicals” and “allergic to bee sting”. Smoking habits were grouped into the categories “non-smoker”, “1-9 cigarettes per day” and “10 or more cigarettes per day”. The patients’ own assessments of their health were registered as “healthy” or “unhealthy”. Drugs were classified according to the ATC code and the Swedish Medicines Compendium for Physicians (FASS). In cases in which a single chemical substance belonged to two or more ATC codes, the code for a systemic route of administration was selected over topical administration. A visual analogue scale (VAS) was used to register symptoms from the oral mucosa. For statistical purposes, the scores were divided into four groups: “no symptoms”, “VAS <30”, “VAS ?30”, and “VAS ?60”.

-Agreement between GP and oral medicine specialists in diagnosis

In the cases in which a tentative diagnosis was established by GP, the oral medicine specialists (OMS; MJ and UM) made their diagnosis from the corresponding clinical photos. The diagnoses made by the GP were then compared with those made by the OMS and the level of agreement was determined. The comparison was classified as follow: “OMS agreed with the GP”, “OMS disagreed with the GP”, and “one of the OMS agreed with the GP but disagreed with the other OMS”.

-Statistical analysis

For statistical purposes, a group of 1,029 patients without OML was compared to the patients with OML (n=1031). The groups were compared for all of the variables registered during the clinical examination (see above) in order to identify associations between the different parameters and OML. Moreover, groups of diagnoses including 20 patients or more were compared to the control group in order to see if there was a correlation with any of the registered variables. Possible correlation between symptoms that were awarded a VAS score ?30 and any of the aforementioned variables was also explored. Fisher’s exact test was used in the analysis and a P-value <0.05 was regarded as statistically significant. The statistical analysis was performed using the Statistical Analysis Software version 9.2 (SAS Institute Inc. Cary, NC, USA).

## Results

-Symptoms, disease history and demographics of the study population

A total of 141 patients (14.8%) with OML reported subjective symptoms from the oral mucosa. Of these, 43 patients (4.5%) scored their symptoms <30, 65 patients (6.8%) scored their symptoms ?30, and 28 patients (2.6%) scored their symptoms ?60 in a VAS. Thirty-three patients (3.5%) presented with subjective symptoms but no score in a VAS was registered. A group of 11 patients without any clinically detectable OML also presented with subjective symptoms from the oral mucosa.

The groups were not matched for age or sex when OML patients were compared with the controls. The OML group comprised more women (P<0.05). The control group reported more malignant neoplasms than did the OML patients (P<0.05). Conversely, OML patients reported more ischemic heart diseases and chronic lower respiratory diseases (P<0.05). A statistically significant difference was also found for smoking habits, with a higher prevalence in the control group (P<0.05). There was no statistically significant difference between the groups for self-reported illness. Four hundred and seventy-six (46%) patients with OML were using at least one regular systemic medication at the time of the initial examination compared to 452 (44%) in the control group (n.s.). Use of regular medication was found more often among men than women in both groups, and 53.2% of the patients with OML used daily medication compared to 61.7% of those in the control group (P<0.01). However, no statistically significant differences were found between OML patients and controls in the medication profile according to the 1st level of the ATC code.

The demographics and disease history of the patients with six of the most common OML and the controls are shown in  Table 1. In general, patients with aphthous ulcers reported more allergies than did the controls (P<0.05). Also, the prevalence of geographic tongue (P<0.05) and snuff lesions (P<0.001) was significantly higher among males than among females. Smoking was more common among patients with leukoplakia than among the controls, although this difference was not statistically significant. Hypertensive diseases were more common among patients with lichenoid lesions, geographic tongue and snuff lesions than among the controls (P<0.05). According to the 1st level of the ATC code, patients with lichenoid lesions (P<0.001), geographic tongue (P<0.01) and fissured tongue (P<0.05) registered a higher consumption of cardiovascular drugs than did the controls.

**Table 1:**
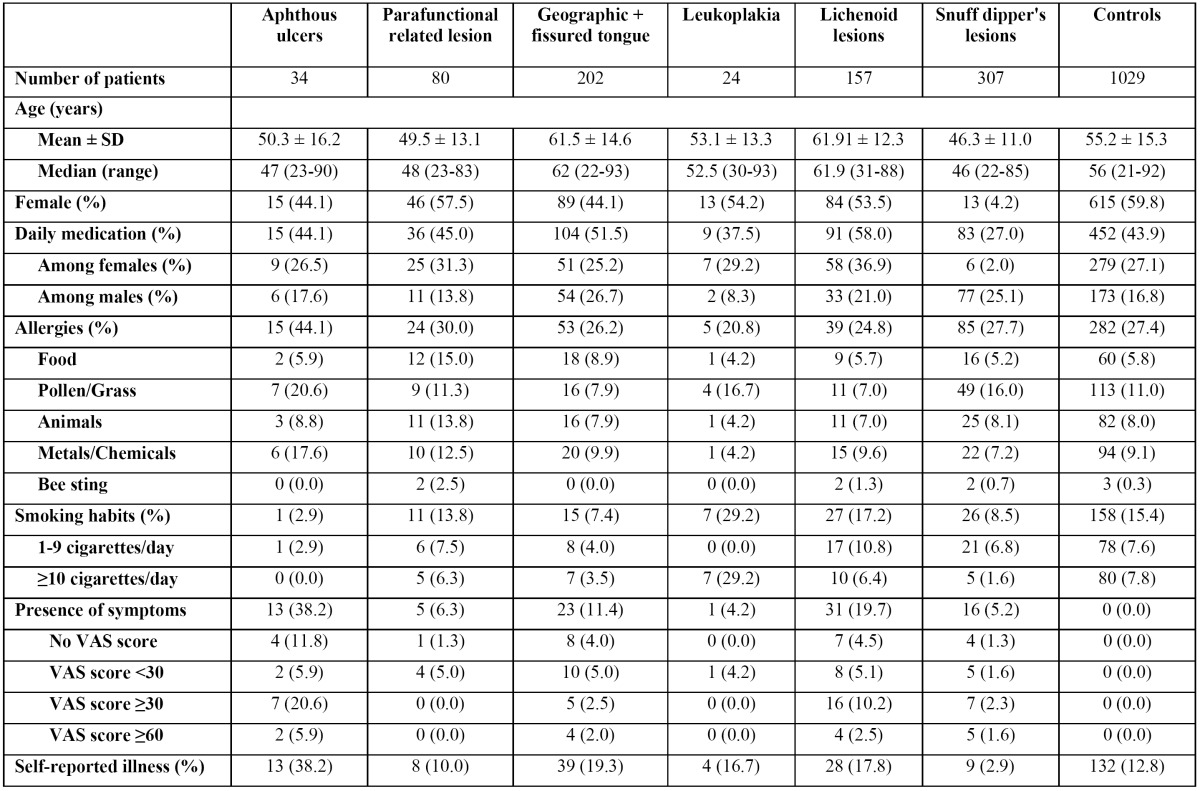
Demographics and medical history of common oral mucosal lesions and controls.

-The prevalence of OML

**Figure 1:**
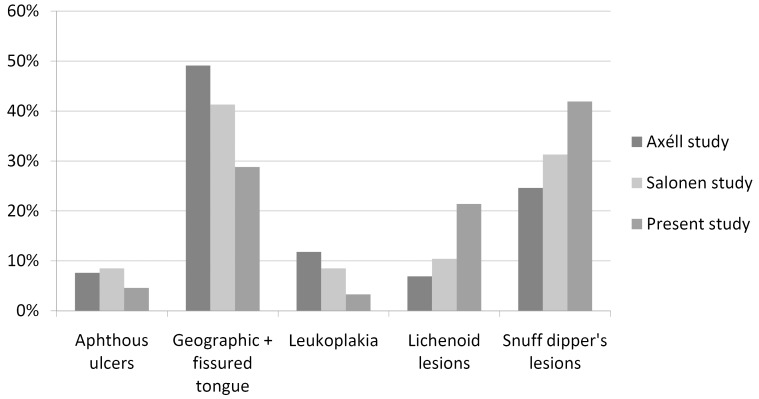
Prevalence of common oral mucosal lesions in the present and previous studies in Sweden.

From the total of 6,448 individuals examined, the GP registered at least one oral condition in 1,031 patients. Of these, 950 (mean age=56.0 years; females n=363), or 14.7% of the patient population, presented with an OML diagnosis according to the classification by Axéll and Axéll et al (12,20). In eighty-one patients the GP registered lesions that could not be categorised according to these classifications. The remaining 5,417 patients presented no OML.  Table 2 shows the prevalence of OML in the study population and compares the estimates with those from the two previous studies conducted in Sweden. Snuff dipper’s lesion, lichenoid lesions, geographic and fissured tongue, aphthous ulcers and leukoplakia were compared with the corresponding diagnoses from the two previous epidemiological studies conducted in Sweden (Fig. 1).

**Table 2:**
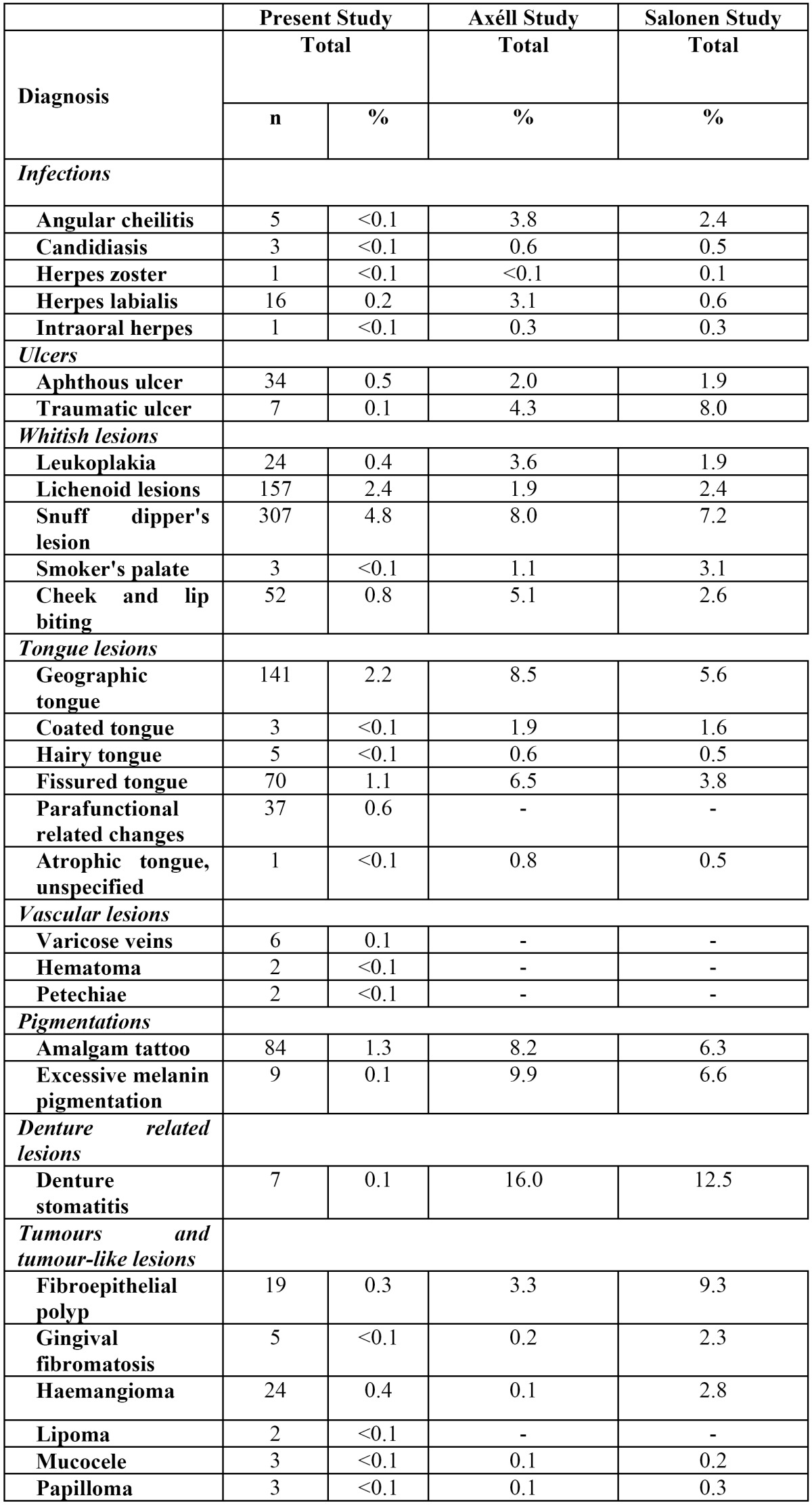
Prevalence of oral mucosal lesions in 6448 Swedish adult patients.

Although parafunctional related lesions were among the most prevalent OML, they were excluded from the analysis since some of these lesions, such as impressions on the lateral border of the tongue, were not reported in the studies by Axéll and Salonen et al (12).

-Agreement in diagnosis of OML

In 85% of the cases (n=803) the OMS and GP were in agreement over the diagnosis, in 14% there was disagreement between the OMS and the GP, and in 1% there was disagreement between the OMS. The level of agreement between the OMS and GP for the twelve most common OML diagnoses is shown in  Table 3. The highest rate of agreement between OMS and GP was found for geographic tongue, followed by snuff lesions and fissured tongue. By contrast, herpes lesion was the diagnosis that GP most frequently misdiagnosed. The OMS were also able to establish a diagnosis in 118 cases where GP had made no diagnosis.

**Table 3:**
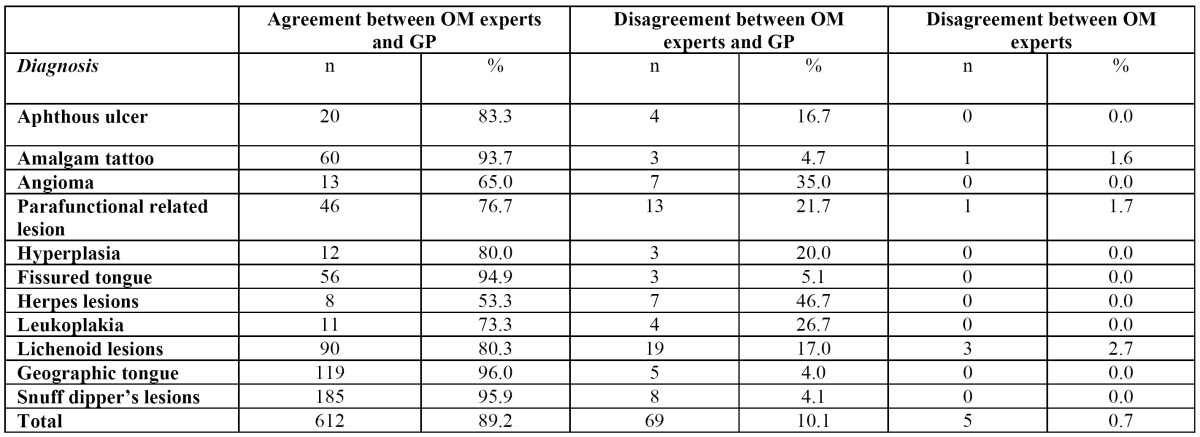
Agreement between general practitioners and oral medicine specialists in the diagnosis of oral mucosal lesions.

## Discussion

The main objective of this study was to investigate the extent to which oral conditions are responsible for pain or discomfort in the general population. No earlier population study has gathered information about subjective symptoms and this is surprising since assessment of QoL represents one of the most important ways of evaluating health (3). Many OML are chronic in nature and they may have a significant impact on the patient’s QoL, not only in terms of physical functioning but also in terms of their psychological and social effects (2).

A recent study found a good correlation between two QoL instruments (COMDQ and OHIP-14) and VAS (1). One per cent of our patients reported a VAS ?30, and if this is extrapolated to the whole Swedish population over 20 years of age, this would mean that approximately 70,000 individuals in Sweden are suffering from significant discomfort from the oral mucosa. As might be expected, the lesions that may involve ulcerations, such as aphthous stomatitis and lichenoid reactions, were given the highest VAS scores.

Several studies have reported the prevalence of OML (12-17,21-27), but there are differences in how registration of OML was carried out. Several studies have focused on specific lesions that may be associated with, for example, an increased risk of cancer or on lesions associated with tobacco or betel use (15,21,25). Some studies describe the prevalence in the entire adult population (12,14,17,21,23-27), whereas others have selected people aged 65 and over (13) or in two specified age cohorts (15,16). The selection of a particular age group may skew prevalence figures since some lesions are more prevalent in certain age groups. Furthermore, some types of lesions are rare and a large study population is therefore required in order to include them. Consequently, any prevalence figure based on a small sample must be interpreted with caution. There are only four studies that have included more than 10,000 individuals (12,17,25,26). The remaining studies have included populations ranging from 555 (22) to 4,210 (24) individuals. The present study population is therefore the fifth largest reported. To conclude, the methodological differences between studies are considerable, such as patient selection and diagnostic criteria for OML, and this makes direct comparison difficult.

Despite the relatively high number of patients in our study, several diagnoses were not detected. For example, no case of oral cancer was found. This is reasonable given the annual incidence of squamous cell carcinoma in the Swedish population (approximately 600 cases in a population of 9 million). Moreover, our study found no cases of vesiculo-bullous conditions (mucous membrane pemphigoid, pemphigus). This is in accordance with the large population studies conducted by Shulman (17) and Axéll (12), in which none of these diagnoses were found, although “desquamative gingivitis” is described by Axéll in 3 cases. The absence of vesiculo-bullous lesions may be attributed partly to its low prevalence but partly also to the fact that diagnoses of these conditions are usually confirmed by histopathological examination. There are unquestionably also other OML for which a definitive diagnosis requires support from histopathological examination. No biopsies were taken in our study and this is clearly a limitation. However, this is also true of most of the large prevalence studies. Biopsies were only performed in four studies (12,14,26,27) and then mainly on lesions that were suspected of malignancy.

Statistical analysis of the demographics and medical history ( Table 1) was only performed when a diagnosis was registered for more than 20 patients, since it was considered statistically irrelevant to analyse diagnoses that were made for fewer patients. Amalgam tattoo and haemangioma were excluded from the analysis, since no correlation with medical conditions was expected. Relationships between specific OML, including conditions such as aphthous stomatitis, oral lichen planus, erythema multiforme and orofacial granulomatosis, and certain general diseases/drugs have often been examined. By contrast, information about medical history is seldom described in prevalence studies of OML. Demographic data and use of drugs were registered by Axéll but they were not presented or discussed in terms of their correlation with specific OML. Mumcu *et al.* (23) recorded “systemic diseases” and “medication use” but no details were provided. The continuous registration of formalized information in our computerised system, MedView, enables such analyses. To the best of our knowledge, we are the first to correlate medical data with a wide variety of OML. Although no significant correlations were found in this study, the potential benefits of being able to analyse such correlations should be emphasized.

Crucial to any epidemiological study is the reliability of its data, regarding both the general medical information and the registered diagnoses. In our study the general medical information was obtained through interviews performed by the GP. Also of importance is the reliability of registered OML diagnoses. Some studies assess prevalence according to data obtained from examinations by specialists (12-14,22,27). Other studies have been conducted by GP in order to collect large quantities of data in less time (15-17,21,24-26). Most studies have included training and calibration in order to enhance the quality of registrations. However, only a few studies have included analysis of the degree of agreement between observers and specialists (15,16,27), although this is advisable for ensuring the quality of obtained results. We also found that agreement between the GP’ and OMS’ diagnoses varied depending on the type of condition and this suggests that reliability was higher for some diagnoses than for others. Finally, the OMS detected some lesions in images where GP did not register any OML. This may be explained either by the fact that the GP had noted a lesion but been unable to make a diagnosis or that some lesions were simply overlooked on clinical examination.

When comparing our results with those of previous studies it seems reasonable to compare them to the Swedish population studies by Axéll (12) and Salonen *et al.* (27). Both of these studies were performed by experienced specialists and agreement between the findings of each was substantial. There are clearly major differences between the prevalence figures found in the two previous Swedish population studies and those found in ours. Generally our prevalence rates are lower, which may be explained by differences in methodology and the fact that the disease panorama has changed. Axéll’s and Salonen’s studies have considerable methodological similarities since one of the authors participated in both studies. In the present study, GP may have under-reported if they only registered the more accentuated lesions. For example, no snuff-related lesions were observed by the GP in 47 patients who used three or more packs of snuff per week, although it is highly unlikely that these patients had a completely healthy oral mucosa. It is possible that an oral medicine expert would have found a snuff dipper’s lesion.

If the percentage distribution of the five most frequently reported changes is analysed, Salonen’s distribution falls between Axéll’s and that of the present study. The lower prevalence of snuff-related lesions and leukoplakia makes sense since it corresponds with consumption changes over time. In Sweden for the period 1990-2007, the prevalence of smoking decreased among men from 26% to 16% and among women from 27% to 18% (28). The use of snuff increased among men from 20% to 28.6% and among women from 1.4% to 8.4% (28). The prevalence of lichenoid lesions remained roughly the same in all three studies and this may be explained by the fact that the prevalence of lichenoid lesions has not changed significantly over time.

The analysis of the correlation between diagnoses made by the GP and those made by the OMS showed that previously trained GP could contribute significantly to the collection of large quantities of reliable and accurate clinical data that could be used for epidemiological analysis in the oral medicine field. However, it should be stressed that although the GP were trained prior to the start of this study, as Splieth et al. (24) described, GP should ideally receive continuous training and be monitored throughout the entire study to optimize reliability.
